# Prognostic significance of the expression of metastasis-associated in colon cancer-1 in gynecologic cancers and breast cancer: A protocol for systematic review and meta-analysis

**DOI:** 10.1097/MD.0000000000024255

**Published:** 2021-02-26

**Authors:** Lijun Wang, Liying Fan, Hongyan Xu, Haiyuan Jiang

**Affiliations:** a Department of Obstetrics and Gynecology, Chun’an First People's Hospital, Chun’an; b Department of Obstetrics and Gynecology, Xiasha Hospital of Zhejiang Provincial Hospital of Traditional Chinese Medicine, Hangzhou, Zhejiang, China.

**Keywords:** breast cancer, gynecologic cancers, meta-analysis, metastasis-associated in colon cancer-1, prognosis

## Abstract

**Background:**

The prognostic role of the expression of metastasis-associated in colon cancer-1 (MACC1) in gynecologic cancers and breast cancer remains unclear. The aim of this systematic review and meta-analysis was to determine the prognostic significance of MACC1 expression in gynecologic cancers and breast cancer.

**Materials and methods:**

PubMed, Web of Science and Embase were comprehensively searched up to February 9, 2020. Studies focusing on the relationship between the expression of MACC1 and prognosis in gynecologic cancers and breast cancer were included into the analysis. Pooled hazard ratio (HR) or odd ratio with 95% confidence interval (CI) was used to estimate the prognostic value of the expression of MACC1.

**Results:**

A total of 1,811patients with gynecologic cancers or breast cancer were included into the analysis. Patients with high expression of MACC1 tended to suffer a shorter overall survival (HR = 2.76, 95%CI = 2.12–3.59, *P* < .01) and recurrence-free survival (HR = 2.37, 95%CI = 1.44–3.90, *P* < .01) compared to those with low expression of MACC1. High expression of MACC1 was significantly associated with worse tumor differentiation (*P* = .04), more advanced FIGO stage (*P* < .01) and earlier lymph node metastasis (*P* < .01) compared to low expression of MACC1.

**Conclusion:**

Compared to low expression of MACC1, high expression of MACC1 predicts a worse prognosis of gynecologic cancers and breast cancer. The expression of MACC1 can serve as a prognostic indicator of gynecologic cancers and breast cancer.

## 1 Introduction

Gynecologic cancers and breast cancer have become the leading cause of death for women worldwide.^[1]^ Despite great development of diagnosis and treatment, patients with gynecologic cancers or breast cancer surfer from a disappointing prognosis, especially those at advanced clinical stage.^[2,3]^ Hence, many investigators try to identify promising prognostic indicators to serve as potential therapeutic targets and improve the clinical decision-making.^[4–6]^

Metastasis-associated in colon cancer-1 (MACC1), located on chromosome 7 at position 7p21.1, was proved to regulate the hepatocyte growth factor/Met signaling pathway in colon cancer.^[7,8]^ Previous researches showed the dysregulated expression of MACC1 contributed to the carcinogenesis, invasion, and migration of human tumors.^[9]^ The dysregulated expression of MACC1 has been proved to be associated with the prognosis of several digestive cancers, including colon cancer,^[10]^ gastric cancer^[11]^ and hepatocellular carcinoma.^[12]^ Recently, accumulating evidence showed MACC1 may play an important role in the progression of gynecologic cancers and breast cancer, and MACC1 may have the potential capacity to predict the prognosis of these patients with gynecologic cancers or breast cancer.^[13–22]^*Yu et al.* analyzed 207 cases diagnosed as ovarian cancer and found high expression of MACC1 was associated with shorter overall survival (OS) compared to low expression of MACC1 [hazard ratio (HR)=4.06, *P* < .01].^[20]^*Zhou* et al study showed, compared to low expression of MACC1, high expression of MACC1 was related to worse OS in cervical cancer (HR=2.99, *P* = .04).^[22]^ Huang et al also found high expression of MACC1 might predict a worse OS compared to low MACC1 expression in breast cancer (HR = 3.19, *P* < .01).^[15]^ Currently, despite diverse articles revealed the potential link between the expression of MACC1 and prognosis of gynecologic cancers and breast cancer, the prognostic value of MACC1 expression in gynecologic cancers and breast cancer remains contradictory, which may be attributed to the limited sample size and imperfect design.^[13–23]^ Here, we performed this systematic review and meta-analysis to determine the prognostic value of MACC1 expression in gynecologic cancers and breast cancer.

## 2 Materials and methods

Ethical approval and informed consent were unnecessary because no data of individuals was used in the analysis. This study was conducted strictly according to Preferred Reporting Items for Systematic Reviews and Meta-Analyses.^[24]^

### 2.1 Eligibility criteria

The included studies should meet the following criteria:

(1) Participant: patients diagnosed as any gynecologic cancers or breast cancer;
(2) Intervention: high or positive expression of MACC1;
(3) Control: low or negative expression of MACC1;
(4) Outcome: OS, progression-free survival (PFS), recurrence-free survival (RFS), disease-free survival (DFS) and clinicopathological parameters;
(5) Study design: retrospective or prospective studies.

Exclusion criteria were as follows: not gynecologic cancers or breast cancer, cell or animal experiments, reviews, case reports, letters, insufficient data or duplicated patients.

### 2.2 Literature search and study selection

PubMed, Web of Science and Embase were comprehensively research on February 9th, 2020 using the following strategy: (“MACC1” OR “Metastasis-associated in colon cancer-1”) AND (“carcinoma” OR “cancer” OR “tumor” OR “neoplasm”) AND (“survival” OR “prognosis”). The references of retrieved studies were also checked to avoid missing relevant studies. Then, study selection was performed according to the eligibility by 2 investigators independently, and any disagreement would be solved by group discussion.

### 2.3 Data extraction

Data extraction was conducted independently by 2 investigators using a prepared template, and any disagreement was resolved by reaching a consensus on all contents. Following items were extracted from included studies: first author, publication year, retrospective design, sample size, patients with high or low expression of MACC1, detection method of the expression of MACC1, source of sample, cancer type, outcomes, and analysis model of OS. As for prognostic variables (eg, OS, RFS, PFS and DFS), HR with 95% confidence interval (CI) was directly extracted from articles published or indirectly extracted as described by Tierney et al.^[25]^ Besides, clinicopathological parameters were also extracted, including tumor differentiation, tumor size, FIGO stage, ascites, and lymph node metastasis.

### 2.4 Risk of bias of included studies

Risk of bias of included studies was evaluated using the Newcastle-Ottawa Scale score, in which the scores varied from 0 to 9, and a score greater than 5 was regarded as low risk of bias.^[26]^

### 2.5 Statistical analysis

All analyses in this study were conducted by Review Manager 5.3 software (Cochrane Collaboration, London, UK) and Stata 12.0 (StataCorp, College Station, TX). Pooled HR with corresponding 95% CI was utilized to explore the association between MACC1 expression and prognosis in gynecologic cancers and breast cancer. The association between the expression of MACC1 and clinicopathological parameters was described using odds ratio (OR) with corresponding 95% CI. Cochran *Q* and Higgins *I*^2^ tests were applied to assess the heterogeneity among included studies. Heterogeneity was considered obvious as *I*^2^ > 50% and *P* < .10, and a random-effect model was used; otherwise, a fixed-effect model was applied. To comprehensively evaluate the association between the expression of MACC1 and OS, subgroup analysis was conducted. Begg and Egger tests were performed to evaluate the publication bias, and sensitivity analysis was employed to confirm the reliability of the results using Stata 12.0. A 2-side *P* value less than .05 indicated that the association between the expression of MACC1 and prognostic outcomes was statistically significant.

## 3 Results

### 3.1 Literature search and study selection

**Figure 1:**
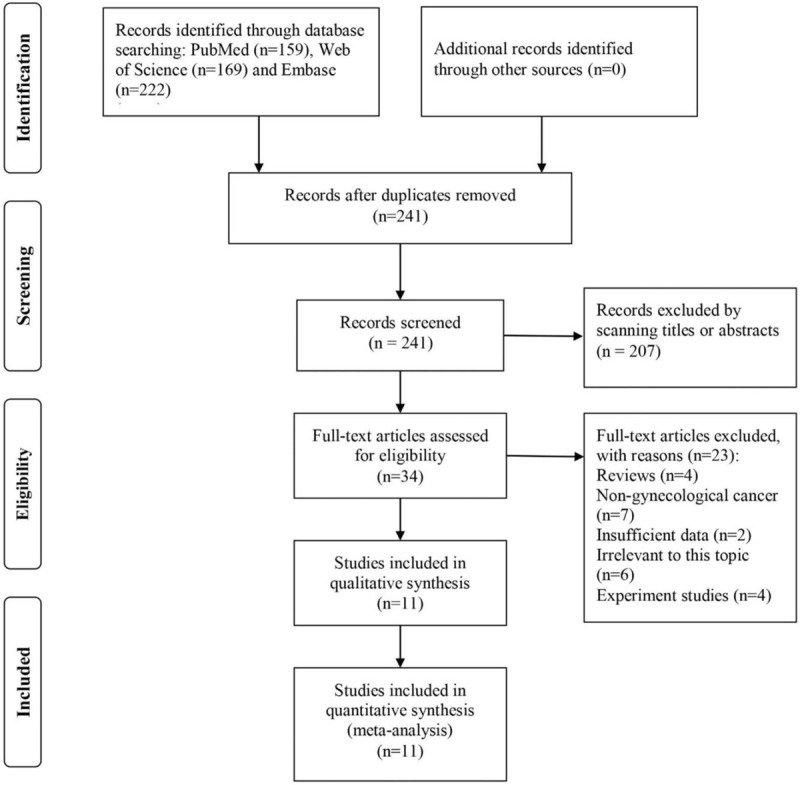
Flow chart of literature search and study selection.

As shown in Figure 1, a total of 550 articles were retrieved from 3 common databases, and 241 articles remained for further examination after the removal of duplications. Among 241 articles, 207 studies were directly excluded by scanning the titles or abstracts. For the rest articles, full-text of each study was evaluated and 23 studies were excluded for following reasons: review type (n = 4), not gynecologic cancers or breast cancer (n = 7), insufficient data (n = 2), irrelevant to this topic (n = 6) and experiment studies (n = 4). Finally, a total of 11 studies were included into this systematic review and meta-analysis.^[13–23]^

### 3.2 Characteristics of included studies

The characteristics of included studies were listed in Table 1. A total of 11 retrospective studies containing 1,811 patients with gynecologic cancers or breast cancer were included into this research.^[13–23]^ Especially, Huang et al study contained 2 independent clinical cohorts.^[15]^ Nine studies were conducted in China,^[13–16,18–22]^ 1 study was conducted in Germany^[17]^ and 1 study was conducted in Bosnia and Herzegovina.^[19]^ The expression of MACC1 was detected using immunohistonchemistry in 6 studies,^[13–16,20,22]^ real-time quantitative polymerase chain reaction in 3 studies^[17,18,21]^ and other methods in 2 studies.^[23]^ The sample was obtained from tumor tissue in 9 studies^[13–16,18–22]^ and preoperative serum in 2 studies.^[17,23]^ Four types of cancer were investigated, including ovarian cancer,^[16,17,20,21]^ breast cancer,^[15,19,23]^ cervical cancer^[14,18,22]^ and endometrial cancer.^[13]^ The FIGO stage was reported in all included studies.^[13–23]^ For outcomes, 10 studies reported the clinicopathological parameters,^[13–16,18–23]^ 7 studies reported the OS^[13–17,19,20]^ and 6 studies reported the RFS, PFS, or DFS.^[13–15,17,19,23]^ The association between the expression of MACC1 and OS was evaluated using the multivariate analysis in 4 studies^[14,15,20,22]^ and using the univariate analysis in 2 studies.^[13,17]^ The Newcastle-Ottawa Scale score of all included studies was larger than 5, indicating no obvious risk of bias among studies.^[13–23]^

**Table 1:** Characteristics of included studies.

Study	Country	Study design	Detection method	Source	MACC1 expression (n) (total/high/low)	Cancer type	FIGO stage (I+II/III+IV)	Outcome	Analysis model	NOS
Huang et al 2013^[1,15]^	China	R	IHC	Tumor tissue	245/136/109	Breast cancer	172/73	CP, RFS, OS	M	8
Huang et al 2013^[2,15]^	China	R	IHC	Tumor tissue	185/111/74	Breast cancer	101/84	CP, OS	M	8
Guo et al 2014^[14]^	China	R	IHC	Tumor tissue	104/51/53	Cervical cancer	71/33	CP, RFS, OS	M	8
Li et al al 2014^[16]^	China	R	IHC	Tumor tissue	47/33/14	Ovarian cancer	10/37	CP	NA	6
Zhou et al 2015^[22]^	China	R	IHC	Tumor tissue	181/96/85	Cervical cancer	130/51	CP, OS	M	7
Chen et al 2016^[13]^	China	R	IHC	Tumor tissue	158/68/90	Endometrial cancer	136/22	CP, RFS, OS	U	8
Tang et al 2016^[23]^	China	R	ELISA	Serum	378/253/125	Breast cancer	270/108	CP, DFS	NA	7
Yu et al 2017^[20]^	China	R	IHC	Tumor tissue	207/126/81	Ovarian cancer	106/101	CP, OS	M	8
Prguda-Mujic et al 2018^[19]^	Bosnia and Herzegovina	R	WB	Tumor tissue	105/30/75	Breast cancer	T1/T2/T3–4, 23/69/11; N0/N1,67/38	CP, OS, DFS	NA	8
Link et al 2019^[17]^	Germany	R	RT–qPCR	Serum	79/NA/NA	Ovarian cancer	16/63	PFS, OS	U	7
Meng et al 2019^[18]^	China	R	RT–qPCR	Tumor tissue	57/29/28	Cervical cancer	27/30	CP	NA	6
Zhang et al 2019^[21]^	China	R	RT–qPCR	Tumor tissue	65/33/32	Ovarian cancer	12/53	CP	NA	6

CP = clinicopathological parameters, DFS = disease-free survival, ELISA = enzyme linked immunosorbent assay, FIGO = International Federation of Gynecology and Obstetrics, IHC = immunohistonchemistry, M = multivariate, MACC1 = metastasis-associated in colon cancer-1, NA = not available, NOS = Newcastle-Ottawa Scale, OS = overall survival, PFS = progression-free survival, R = retrospective, RFS = recurrence-free survival, RT–qPCR = real-time quantitative polymerase chain reaction, U = univariate, WB = western blot.

### 3.3 Association between the expression of MACC1 and OS

**Figure 2:**
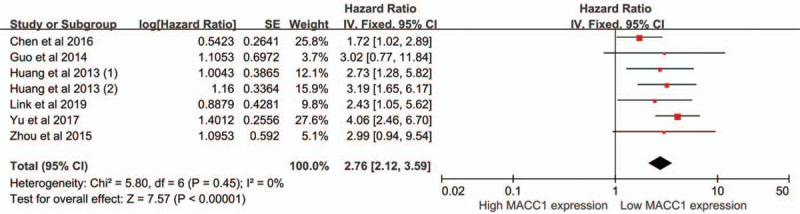
Meta-analysis of association between MACC1 expression and OS.

A total of 6 studies containing 7 cohorts were included into the analysis^[13–15,17,20,22]^ (Fig. 2). There was no heterogeneity among studies, as a result, a fixed-effect model was used (*I*^2^ = 0%, *P*_heterogeneity_ = .45), and patients with high expression of MACC1 tended to have a shorter OS compared to those with low expression of MACC1 (HR = 2.76, 95%CI = 2.12–3.59, *P* < .01). The subgroup analysis also showed high expression of MACC1 was an unfavorable prognostic factor of gynecologic cancers and breast cancer (*P* < .05) (Table 2).

**Table 2:** Subgroup analysis of association between the MACC1 expression and OS.

Variables	Included cohort (n)	HR 95%CI	*P*	*I*^2^ (%)	*P*_heterogeneity_	Model
Country						
China	6	2.80 (2.12, 3.70)	<.01^¶^	12	.34	Fixed
Others	1	2.43 (1.05, 5.62)	.04^¶^	NA	NA	Fixed
Sample size (n)						
≤158	3	1.98 (1.30, 3.01)	<.01^¶^	0	.64	Fixed
>158	4	3.43 (2.45, 4.81)	<.01^¶^	0	.83	Fixed
Cancer type						
Ovarian cancer	2	3.55 (2.31, 5.45)	<.01^¶^	6	.30	Fixed
Endometrial cancer	1	1.72 (1.02, 2.89)	.04^¶^	NA	NA	Fixed
Cervical cancer	2	3.00 (1.24, 7.27)	.01^¶^	0	.99	Fixed
Breast cancer	2	2.98 (1.81, 4.90)	.01^¶^	0	.76	Fixed
Detection Method						
IHC	6	2.80 (2.12, 3.70)	<.01^¶^	12	.34	Fixed
Others	1	2.43 (1.05, 5.62)	.04^¶^	NA	NA	Fixed
Analysis model						
Univariate	2	1.89 (1.22, 2.94)	<.01^¶^	0	.49	Fixed
Multivariate	5	3.41 (2.45, 4.73)	<.01^¶^	0	.92	Fixed

CI = confidence interval, HR = hazard ratio, IHC = immunohistonchemistry, MACC1 = metastasis-associated in colon cancer-1, NA = not available, OS = overall survival.

¶ *P* < .05 indicating the significant association between MACC1 expression and OS.

### 3.4 Association between the expression of MACC1 and RFS/PFS/DFS

**Figure 3:**
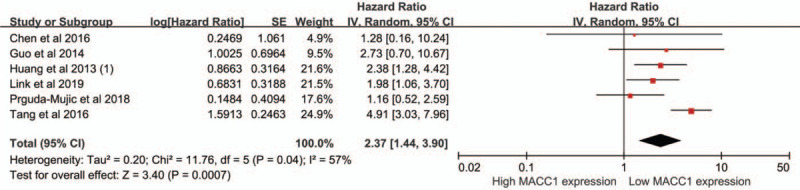
Meta-analysis of association between MACC1 expression and RFS.

Three studies reported the RFS,^[13–15]^ 2 studies reported the DFS^[19,23]^ and 1 study reported the PFS,^[17]^ and all of them were included into the analysis of RFS. A random-effect model was applied for the obvious heterogeneity (*I*^2^ = 57%, *P*_heterogeneity_ = .04). High expression of MACC1 was significantly associated with shorter RFS/PFS/DFS in gynecologic cancers and breast cancer (HR = 2.37, 95%CI = 1.44–3.90, *P* < .01) (Fig. 3).

### 3.5 Association between the expression of MACC1 and clinicopathological parameters

As listed in Table 3, high expression of MACC1 was significantly associated with worse tumor differentiation (OR = 2.23, 95%CI = 1.05–4.74, *P* = .04), more advanced FIGO stage (OR = 3.53, 95%CI = 2.71–4.60, *P* < .01) and earlier lymph node metastasis (OR = 2.87, 95%CI = 2.19–3.77, *P* < .01) when compared to low expression of MACC1. No obvious association between the expression of MACC1 and clinicopathological parameters was observed in terms of age (*P* = .84), tumor size (*P* = .56) or ascites (*P* = .79).

**Table 3:** Association between the MACC1 expression and clinicopathological parameters.

Variables	Cohort (n)	Patients (n)	OR 95%CI	*P*	*I*^2^ (%)	*P*_heterogeneity_	Model	Begg test	Egger test
Age (old/young)	9	1249	1.02 (0.81, 1.29)	0.84	0	.6	Fixed	0.47	0.05
Differentiation (*poor/ well or moderate*)	6	762	2.23 (1.05, 4.74)	0.04^¶^	76	<.01	Random	0.13	0.15
Tumor size (large/small)	2	264	1.16 (0.70, 1.92)	0.56	50	.16	Fixed	NA	NA
FIGO stage (*III+IV/I+II*)	9	1249	3.53 (2.71, 4.60)	<0.01^¶^	0	.65	Fixed	0.18	0.05
Ascites (yes/no)	2	272	1.23 (0.27, 5.58)	0.79	81	.02	Random	NA	NA
LNM (yes/no)	8	1202	2.87 (2.19, 3.77)	<0.01^¶^	36	.14	Fixed	0.11	0.06

CI = confidence interval, FIGO = International Federation of Gynecology and Obstetrics, LNM = lymph node metastasis, MACC1 = metastasis-associated in colon cancer-1, NA = not available, OR = odd ratio.

¶ *P* < .05 indicating the significant association between MACC1 expression and clinicopathological parameters.

### 3.6 Sensitivity analysis

**Figure 4:**
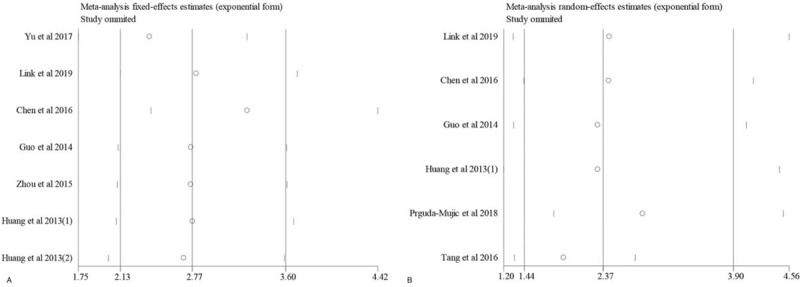
Sensitivity analysis of association between MACC1 expression and OS or RFS (A, OS; B, RFS).

The reliability of the association between the expression of MACC1 and OS (Fig. 4A) or RFS (Fig. 4B) was checked by sensitivity analysis.

### 3.7 Publication bias

**Figure 5:**
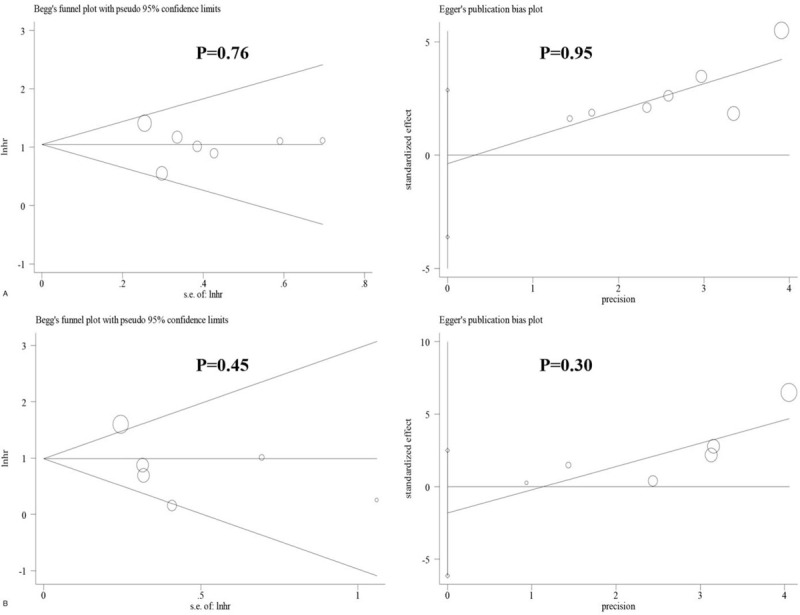
Publication bias of association between MACC1 expression and OS or RFS (A, OS; B, RFS).

There was no obvious publication bias across studies with respect to the association of the expression of MACC1 with OS (Begg test, *P* = .76; Egger test, *P* = .95) (Fig. 5A), RFS (Begg's test, P = 0.45; Egger test, *P* = .30) (Fig. 5B) or clinicopathological parameters (Table 3).

## 4 Discussion

Plenty of studies have showed the dysregulated expression of MACC1 contributed to the tumor progression and could predict the prognosis of gynecologic cancers and breast cancer, otherwise, definite conclusion has not been obtained for the small sample size and contradictory results among previous studies.^[13–23]^ In the current study, we performed a systematic review and meta-analysis to determine the prognostic role of MACC1 expression in gynecologic cancers and breast cancer, and our results showed high expression of MACC1 was significantly associated with shorter OS and RFS compared to low expression of MACC1. The subgroup analysis stratified by the country, sample size, cancer type, detection method and analysis model of OS confirmed the significant association between the expression of MACC1 and OS. Moreover, patients with high expression of MACC1 tended to suffer from worse tumor differentiation, more advanced clinical stage and earlier lymph node metastasis compared to those with low expression of MACC1. In a word, our results showed high MACC1 expression is an unfavorable prognostic factor of patients with gynecologic cancers or breast cancer, and the MACC1 may be a potential therapeutic target of gynecologic cancers and breast cancer.

Although a great number of studies have reported the prognostic role of MACC1 expression in gynecologic cancers and breast cancer, the underlying mechanism remains indistinct.^[13–23]^ Chen et al study showed the overexpression of miR-23b can reduce the expression of MACC1, induce the G1 phrase arrest, and suppress the cell proliferation in endometrial cancer.^[13]^ Li et al found that MACC1 may promote the tumor progression via the signal pathway of hepatocyte growth factor/c-Met in ovarian cancer.^[16]^ Zhang et al study showed the expression of miR-338–3p is negatively correlated to the expression of MACC1 in ovarian cancer.^[21]^ Zhang et al study showed MACC1 can improve the chemosensitivity of cisplatin in ovarian cancer cells through the ERK1/2 signaling pathway on glycoprotein and its downstream apoptosis proteins.^[27]^ Meng et al study indicated that the expression of MACC1 may be regulated by the miR-877, and MACC1 can restore the miR-877 overexpression-mediated the suppression of proliferation and invasion of cervical cancer cells.^[18]^ Yu et al found that long non-coding RNA HCP5 can promote the progression of cervical cancer by regulating MACC1 via the suppression of microRNA-15a.^[28]^ Wang et al drew the conclusion that miR-485 can suppress the tumor progression via targeting MACC1 and inhibiting the Met/AKT signaling pathway.^[29]^

There were several highlights in the current study. First, to our knowledge, we are first to evaluate the prognostic value of MACC1 expression in gynecologic cancers and breast cancer in the form of systematic review and meta-analysis. Second, we explore the association of MACC1 expression with OS, RFS and clinicopathological parameters, therefore, the analysis of the prognostic value of MACC1 expression in our study is comprehensive and convincing. Third, a large population of 1,811 patients are included into the current study, which can provide the convincing conclusions.

Some limitations should be considered when interpreting our findings. First, all studies have a retrospective design, as a result, selection bias may exist. Second, different methods are used to detect the expression of MACC1, which may reduce the reliability of results. Third, on account of the limited population, we explored the relationship between the expression of MACC1 and prognosis of all types of gynecologic cancers or breast cancer, instead of 1 specific cancer (eg, ovarian cancer, breast cancer or cervical cancer), which may limit the application of our conclusion into clinical practice. Forth, most studies are conducted in China, therefore, it is hard to popularize our findings into other countries.

## 5 Conclusion

High expression of MACC1 predicts shorter OS and RFS compared to low MACC1 expression in gynecologic cancers and breast cancer. High expression of MACC1 is associated with worse tumor differentiation, more advanced FIGO stage and earlier lymph node metastasis in gynecologic cancers and breast cancer. Therefore, MACC1 expression can predict the prognosis of gynecologic cancers and breast cancer. Multicenter prospective trials with large sample size and long follow-up period should be carried out to determine the prognostic value of MACC1 expression in gynecologic cancers and breast cancer in future.

## Acknowledgments

We would like to thank the researchers and study participants for their contributions.

## Author contributions

Study concepts and design: Haiyuan Jiang; Literature search: Lijun Wang and Liying Fan; Data extraction: Lijun Wang and Hongyan Xu; Data analysis: Lijun Wang and Haiyuan Jiang; Manuscript preparation and revision: Lijun Wang and Haiyuan Jiang. All authors have participated sufficiently in the study and approved the final version.

**Conceptualization:** Haiyuan Jiang.

**Data curation:** Lijun Wang, Liying Fan.

**Formal analysis:** Haiyuan Jiang.

**Investigation:** Lijun Wang.

**Methodology:** Lijun Wang, Hongyan Xu, Haiyuan Jiang.

**Resources:** Hongyan Xu.

**Software:** Liying Fan.

**Supervision:** Lijun Wang, Liying Fan, Hongyan Xu, Haiyuan Jiang.

**Validation:** Lijun Wang, Liying Fan, Hongyan Xu.

**Writing – original draft:** Lijun Wang, Liying Fan, Hongyan Xu.

**Writing – review & editing:** Lijun Wang, Liying Fan, Hongyan Xu, Haiyuan Jiang.
